# CRISPR/Cas9-based mutant library screening: the discovery of novel genes regulating immune responses in cotton and rice

**DOI:** 10.3389/fpls.2024.1501092

**Published:** 2024-11-14

**Authors:** Sang Ryeol Park, Seungmin Son

**Affiliations:** National Institute of Agricultural Sciences, Rural Development Administration, Jeonju, Republic of Korea

**Keywords:** CRISPR/Cas9, crop, genetic resources, mutant library screening, new breeding technology, plant immunity

## Abstract

The environmental conditions play a crucial role in determining crop yield, which is essential for ensuring food and nutritional security. However, rapid climate change is exacerbating global environmental stress, leading to severe biotic pressures on crops. Therefore, enhancing crop resilience to pathogens has become one of the most pressing challenges for humanity. Large-scale mutant library screening is the most efficient strategy for identifying numerous genes associated with specific traits. The revolutionary CRISPR/Cas9 system has ushered in a new era in the construction of mutant library. However, its application in crop plants has been relatively scarce compared to mammals, largely due to challenges in accessibility. Fortunately, several research groups have recently developed CRISPR/Cas9-based mutant libraries, successfully identifying a variety of genes involved in crop immunity. In this review, we present an overview and discussion of studies that have generated significant results through the use of CRISPR/Cas9 library screening to identify novel genes associated with resistance to biotic stresses within the field of plant research.

## Introduction

Rapid climate change, driven by environmental degradation and the excessive use of fossil fuels, presents an existential threat to humanity. The detrimental effects of climate change extend to agriculture, significantly jeopardizing crop production, which serves as a critical source of energy and materials for the global population (Rezaei et al., 2023). Crop yields are severely compromised by various pathogens and pest insects. Additionally, climate change tends to promote pathogen proliferation and negatively impacts plant immunity, further intensifying the damage caused by diseases (Velasquez et al., 2018; Subedi et al., 2023; Roussin-Leveillee et al., 2024). Therefore, developing stress-resilient crop varieties and identifying novel genes that enhance stress resilience are crucial for ensuring human survival.

For decades, botanists have used mutant lines to identify novel genes associated with phenotypic traits and elucidate their functions. Large-scale mutant libraries represent important materials for functional genomics and plant breeding (Wang et al., 2013). Mutant libraries have been constructed using traditional methods such as physical mutagenesis (e.g., X-rays, gamma-rays, fast neutrons, and ultraviolet-C radiation), chemical mutagenesis (e.g., ethyl methanesulfonate, ethyl nitrosourea, 1,2:3,4-diepoxybutane, and N-nitroso-N-methylurea), and insertional mutagenesis (e.g., transposons and T-DNA) (Cheng et al., 2017; Ram et al., 2019; Salava et al., 2021). Although mutant libraries generated via traditional methods are valuable, these methods require a lot of time and labor because they generate random mutations and because identifying targeted mutations is difficult (Viana et al., 2019). The development of the revolutionary clustered regularly interspaced short palindromic repeats (CRISPR)/CRISPR-associated nuclease 9 (Cas9)-mediated genome editing technique marked a new era in plant breeding (Son and Park, 2022a). Indeed, some mutant libraries have been constructed using CRISPR/Cas9 tools in crops such as cotton (*Gossypium hirsutum*), maize (*Zea mays*), rice (*Oryza sativa*), soybean (*Glycine max*), and tomato (*Solanum lycopersicum*) (Jacobs et al., 2017; Lu et al., 2017; Meng et al., 2017; Bai et al., 2020; Liu et al., 2020; Ramadan et al., 2021; Chen et al., 2022; Son et al., 2024a; Sun et al., 2024; Wang et al., 2024).

**Figure 1:**
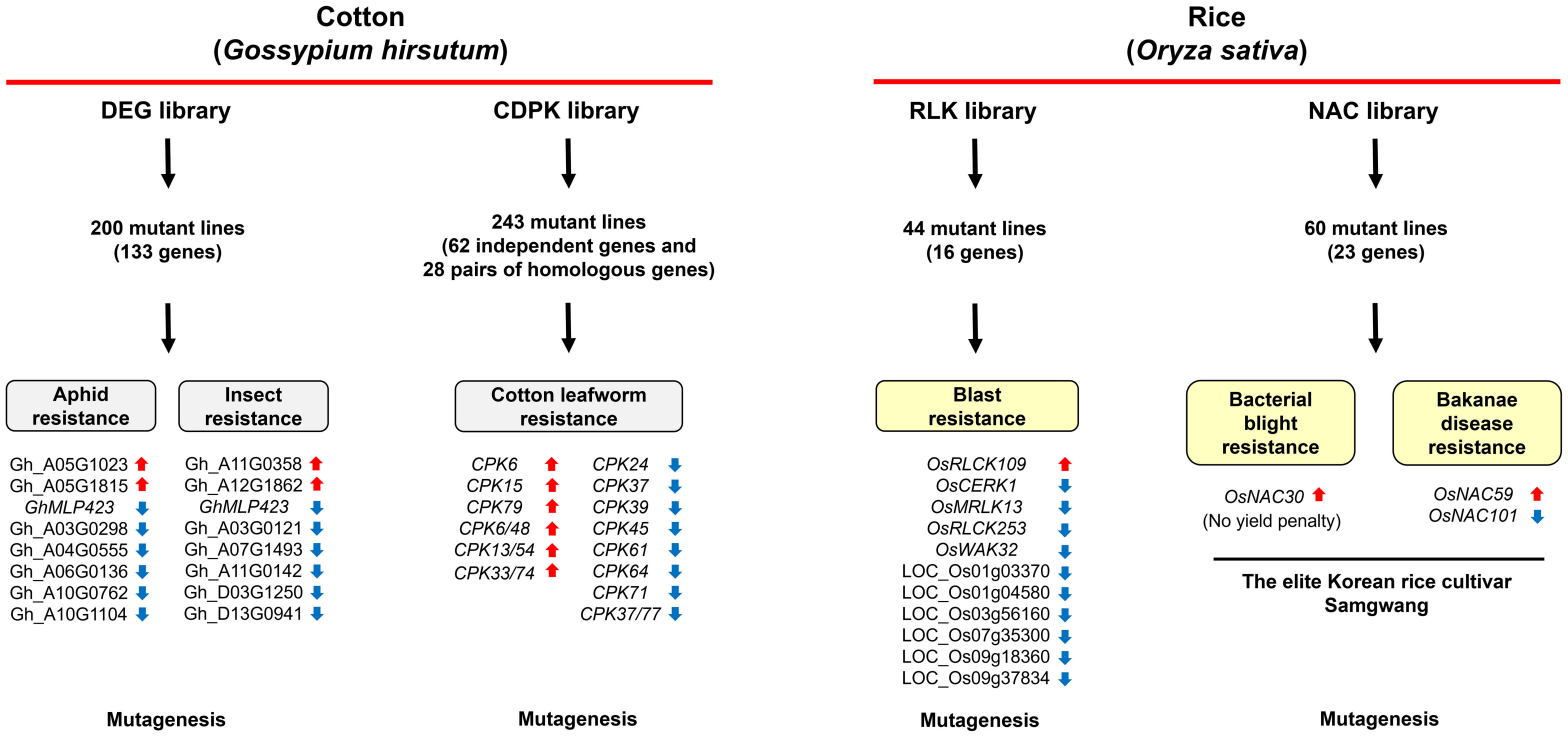
CRISPR/Cas9-mediated mutant library screening for the identification of genes associated with innate immunity in cotton and rice. Using a high-throughput CRISPR/Cas9 system, the differentially expressed gene (DEG) mutant library was constructed for cotton, leading to the identification of 15 genes that significantly influence resistance to various insect pests such as aphids (Sun et al., 2024). Another study utilized CRISPR/Cas9 to develop a cotton calcium-dependent protein kinase (CDPK/CPK) mutant library, identifying six mutant lines with enhanced resistance and eight mutant lines with reduced resistance to *Spodoptera litura* larvae (Wang et al., 2024). In rice, a receptor-like kinase (RLK) mutant library was generated using the FLASH (PCR fragment-length markers for distinguishing gRNA) pipeline for arrayed CRISPR library construction. This approach revealed that mutagenesis of *RECEPTOR-LIKE CYTOPLASMIC KINASE 109* (*OsRLCK109*) heightened rice resistance to *Magnaporthe oryzae*, while mutations in 10 other *RLKs* led to reduced immunity (Chen et al., 2022). Additionally, targeted mutagenesis of rice *NAC* (*no apical meristem, arabidopsis transcription activation factor, and cup-shaped cotyledon*) transcription factor genes using CRISPR/Cas9 revealed that *OsNAC30* mutations in the elite rice cultivar Samgwang enhanced resistance to *Xanthomonas oryzae* pv. *oryzae* without yield penalty, whereas *OsNAC59* mutations increased resistance to *Fusarium fujikuroi* and *OsNAC101* mutations reduced it (Son et al., 2024a). These studies underscore the utility of CRISPR/Cas9-mediated mutant libraries in identifying key genes governing both biotic stress resistance in cotton and rice.

In mammals, genomic approaches utilizing CRISPR library screening to explore target genes and methods for alleviating disease symptoms have been extensively carried out (Srivastava and Pandit, 2023; Chen et al., 2024). However, these efforts have not been actively performed in plants. Although Jacobs et al. generated several tomato leucine-rich repeat subfamily XII gene mutant lines and demonstrated that *S. lycopersicum* FLAGELLIN-SENSITIVE 2.1 (SlFLS2.1) plays a crucial role in the 22–amino acid region of bacterial flagellin (flg22)-induced pathogen-associated molecular pattern (PAMP)-triggered immunity (PTI), this was not based on a library screening (Jacobs et al., 2017). Recently, CRISPR/Cas9-mediated mutant library screening approach was utilized in cotton and rice (Chen et al., 2022; Son et al., 2024a; Sun et al., 2024; Wang et al., 2024), leading to the identification of novel genes associated with resistance to biotic stresses (**Figure 1**).

## Cotton mutant library screening for insect pest resistance

A variety of pests cause substantial reductions in crop yields, resulting in considerable economic losses and jeopardizing global food production (Subedi et al., 2023). Conventional pest control strategies, including the use of chemical pesticides, are associated with environmental risks and can drive the development of resistance in pest populations over time. To address these limitations, Sue et al. present a significant advancement in generating a mutant library of insect-resistant host plants using a high-throughput CRISPR/Cas9 system (Sun et al., 2024). They not only examine the efficacy of this method but also identify a resistance genes, which has the potential to facilitate the development of crops with durable protection against diverse insect species.

To identify cotton genes conferring insect resistance, Sun et al. identified 502 differentially expressed genes (DEGs) and attempted to generate a CRISPR/Cas9-mediated mutant library targeting these genes (Sun et al., 2024). Since these DEGs are implicated in the host plant’s response to insect attacks, they could provide valuable information into the molecular mechanisms governing plant-insect interactions, enabling the identification of potential genetic targets for enhancing pest resistance. Indeed, Sun et al. obtained over 2,000 T_0_ mutant lines and randomly selected 200 independent T_1_ mutant lines, harboring mutations in 133 genes, to assess their insect resistance. The mutagenesis of eight genes (i.e., Gh_A03G1240, Gh_A03G0298, Gh_A04G0555, Gh_A05G1023, Gh_A05G1815, Gh_A06G0136, Gh_A10G0762, and Gh_A10G1104) resulted in altered resistance to aphids, while the mutagenesis of the genes (i.e., Gh_A03G1240, Gh_A03G0121, Gh_A07G1493, Gh_A11G0142, Gh_A11G0358, Gh_A12G1862, Gh_D03G1250, and Gh_D13G0941) led to changes in resistance to chewing pests (Sun et al., 2024). The mutations in Gh_A03G1240, corresponding *to MAJOR LATEX-LIKE PROTEIN 423* (*GhMLP423*), compromised immune responses to both aphids and chewing pests, while *GhMLP423*-overexpressing cotton plants showed enhanced broad-spectrum insect resistance genes (Sun et al., 2024). In subsequent studies, Sun et al. demonstrated that GhMLP423 interacts with the calcium-binding protein (CBP) EPIDERMAL GROWTH FACTOR RECEPTOR SUBSTRATE 15 (GhEPS15) to induce calcium (Ca²^+^) flux, leading to hydrogen peroxide (H_2_O_2_) accumulation, which activates systemic acquired resistance (SAR) (Sun et al., 2024). Therefore, this study establishes a foundational framework for developing cotton with enhanced resilience to a variety of insect pests through the integration of DEG analysis and CRISPR/Cas9-mediated genome editing.

A recent study also reported the development of a calcium-dependent protein kinase (CDPK/CPK) mutant library in cotton. CDPKs play a pivotal role in plant immunity by acting as key mediators of calcium signaling, which is essential for activating various defense responses (Boudsocq and Sheen, 2013). Upon exposure to stress from pathogens or insect pests, plants experience an elevation in intracellular calcium concentrations, which activates CDPKs to initiate a cascade of protective responses. These responses involve the upregulation of defense-associated genes, synthesis of antimicrobial metabolites, and fortification of cell walls to inhibit pathogen penetration (Bacete et al., 2018; Wang and Luan, 2024). By translating calcium signals into downstream immune reactions, CDPKs orchestrate a robust defense, positioning them as key regulators in the plant’s resistance to biotic stresses. Elucidating the specific functions of CDPKs in plant immunity is crucial for advancing strategies aimed at enhancing crop resilience and promoting sustainable agriculture. Therefore, to address the challenge of increased dependence on insect pests and reduce the need for harmful pesticides, Wang et al. generated 518 T_0_ mutant lines using the CRISPR/Cas9 system and subsequently analyzed 243 T_1_ and/or T_2_ mutant plants, involving editing of 62 independent *GhCPKs* and 28 pairs of homologous *GhCPKs*, to assess resistance to larvae of *Spodoptera litura* (Wang et al., 2024). Six mutant lines (i.e., *cpk6*, *cpk15*, *cpk79*, *cpk6/48*, *cpk13/54*, and *cpk33/74*) exhibited enhanced resistance to *S. litura*, whereas eight mutant lines (i.e., *cpk24*, *cpk37*, *cpk39*, *cpk45*, *cpk61*, *cpk64*, *cpk71*, and *cpk37/77*) showed reduced resistance to the pest. Notably, while the *cpk33/74* double mutant line exhibited the highest level of insect resistance, neither the *cpk33* nor the *cpk74* single mutation lines had a significant effect on resistance. Moreover, cotton plants overexpressing *GhCPK33* or *GhCPK74* exhibited increased susceptibility to *S. litura*, indicating that GhCPK33 and GhCPK74 have redundant functions in downregulating the immune response against this insect (Wang et al., 2024). Indeed, GhCPK33 and GhCPK74 inhibited Ca²^+^ flux and jasmonic acid (JA) synthesis, thereby impairing cotton immunity against *S. litura* (Wang et al., 2024). These GhCPKs also interacted with both S-ADENOSYLMETHIONINE SYNTHETASE 1 (GhSAMS1) and GhSAMS2 which are positive regulators of resistance to *S. litura* (Wang et al., 2024). However, the regulatory mechanism of GhCPK33 and GhCPK74 remains to be elucidated.

## Rice RLK mutant library screening for blast resistance

The receptor-like kinase (RLK) superfamily plays important roles in the detection of pathogens and the subsequent activation of defense responses. Although some RLKs, such as receptor-like cytoplasmic kinases (RLCKs), exhibit different subcellular localizations or structural variations, RLKs are typically membrane proteins with extracellular receptor domains (Liu et al., 2024). These membrane-localized proteins function as sensors, recognizing specific PAMPs or damage-associated molecular patterns and triggering a cascade of signaling pathways that enhance the plant’s ability to resist biotic stress (Wu and Zhou, 2013). Upon activation, RLKs initiate various immune responses, including the production of reactive oxygen species, the expression of defense-related genes, and the reinforcement of cell walls, all of which contribute to a robust defense against pathogens (Huang and Joosten, 2024). The variety of RLKs in plants allows for responses to a broad spectrum of pathogens, including bacteria, fungi, and nematodes. Comprehending the specific functions of various RLKs in plant immunity is crucial for deciphering the intricate signaling networks that govern plant defense (Tang et al., 2017; Huang and Joosten, 2024). Moreover, characterizing these receptors can yield critical insights for devising strategies aimed at improving disease resistance in crops, thereby supporting sustainable agricultural practices and enhancing food security. As researchers further explore the roles of RLKs, their importance in plant immunity becomes increasingly evident, underscoring the necessity for innovative strategies to exploit these kinases in crop enhancement.

In rice, with over 1,000 *RLK* genes whose functions are largely unknown, Chen et al. generated a *RLK* mutant library using CRISPR/Cas9 technology to identify novel genes involved in immune responses (Chen et al., 2022). To increase efficiency, they introduced the FLASH (PCR fragment-length markers for distinguishing gRNA) gene editing pipeline to generate an arrayed CRISPR/Cas9 library. As a result, they successfully obtained a rice *RLK* gene mutant library covering 936 *RLK* genes out of a total of 1,072 rice RLK members. Blast disease, caused by the filamentous fungus *Magnaporthe oryzae*, is among the most devastating diseases affecting rice crops (Wilson, 2021). To identify RLK genes involved in defense responses to *M. oryzae*, the causal agent of rice blast, the mutant library was screened. For the initial screening, *RLK* expression levels were assessed, and 14 *RLK* genes exhibiting over 4-fold induction at 72 hours after *M. oryzae* inoculation were selected (Chen et al., 2022). Among these, 9 T_1_ RLK mutant lines (LOC_Os01g03370, LOC_Os01g04580, LOC_Os03g56160, LOC_Os04g24220 [WALL-ASSOCIATED KINASE GENE 32, OsWAK32], LOC_Os04g22470 [MALECTIN/MALECTIN-LIKE RECEPTOR-LIKE KINASE 13, OsMRLK13], LOC_Os07g35300, LOC_Os08g28710 [OsRLCK253], LOC_Os09g18360, and LOC_Os09g37834) demonstrated a diminished immune response to *M. oryzae* (Chen et al., 2022). Therefore, this study not only provides a streamlined method for large-scale gene function analysis but also lays the groundwork for identifying critical RLKs that can be targeted to enhance rice resilience and productivity. The findings have significant implications for global food security and the sustainable improvement of rice, a staple crop for millions.

## Rice NAC mutant library screening for resistance to bacterial and fungal pathogens

In plants, numerous transcription factor families exist, with six major superfamilies—NAC (no apical meristem, arabidopsis transcription activation factor, and cup-shaped cotyledon), APETALA2/ethylene response factor, basic helix-loop-helix, basic leucine zipper, myeloblastosis, and WRKY DNA-binding protein—playing essential roles in mediating innate immunity against a diverse array of pathogens (Tsuda and Somssich, 2015). Especially, the NAC transcription factor superfamily, which plays important roles in plant responses to biotic stress, is associated with multiple stress responses and crop yields. Therefore, the NAC transcription factors are consequently regarded as significant targets in plant breeding (Singh et al., 2021). In rice, there are approximately 146 NAC transcription factors, and previous reports have shown that OsNACs act as both positive and negative regulators of rice immunity against various pathogens. Bacterial blight, caused by the bacterial pathogen *Xanthomonas oryzae* pv. *oryzae* (*Xoo*), and bakanae disease, caused by the fungus *Fusarium fujikuroi*, result in rice yield losses of up to 80% and 50%, respectively, with the potential for more severe damage anticipated due to climate change (Son et al., 2024b). Therefore, identifying the novel genes involved in resistance to these pathogens is crucial for rice breeding. Among the 146 genes, four *OsNAC* genes associated with bacterial blight have been identified, whereas no genes related to bakanae disease had been reported until recently. OsNAC58, OsNAC66, and OsNAC96 act as positive regulators of resistance to *Xoo* (Park et al., 2017; Liu et al., 2018; Wang et al., 2021; Yuan et al., 2021), while OsNAC2, which impairs salicylic acid (SA) synthesis and OsEREBP1, negatively regulate rice immunity to *Xoo* (Zhong et al., 2024). The rice online expression profiles array database and the rice transcription factor phylogenomics database showed almost 35% of *OsNAC* genes responded to *Xoo*; 20 *OsNAC* genes were upregulated and 30 *OsNAC* genes were downregulated in response to this treatment (Chandran et al., 2019), suggesting many OsNACs involved in immune responses. However, *OsNAC* genes regulating rice immunity against *Xoo* and *F. fujikuroi* are largely unknown.

Most recently, Son et al. developed CRISPR/Cas9 plasmids specifically designed to target 146 *OsNAC* genes, which were introduced into the elite rice cultivar Samgwang via Agrobacterium-mediated transformation (Son et al., 2024a). This resulted in the generation of 60 T_1_ homozygous mutant lines harboring mutations in 23 *OsNAC* genes. Given that bacterial blight and bakanae disease are destructive pathogens affecting rice production (Liu and Wang, 2016), disease resistance assays were conducted using homozygous mutant lines to identify the OsNAC transcription factors involved in innate immunity against these pathogens (Son et al., 2024a). The mutagenesis of *OsNAC30* resulted in enhanced resistance to *Xoo* without any yield penalty. Additionally, along with an elevated SA-mediated defense response, which is crucial for plant immunity against *Xoo*, the transcription levels of *SUGAR WILL EVENTUALLY BE EXPORTED TRANSPORTER 13* (*OsSWEET13*) and *OsSWEET14*, which are induced by *Xoo* to promote susceptibility, were reduced in the *osnac30* mutant lines (Son et al., 2024a).

Moreover, OsNAC59 and OsNAC101 were identified as a regulator of innate immunity against bakanae disease. The *osnac59* mutant lines exhibited enhanced resistance to *F. fujikuroi*, indicating that OsNAC59 functions as a negative regulator of resistance to this pathogen (Son et al., 2024a). In *osnac59* mutants, the expression levels of both gibberellin (GA)-related genes, which promote susceptibility to *F. fujikuroi*, and *jasmonate ZIM-domain* (*JAZ*) genes, encoding proteins that inhibit the JA-mediated defense response conferring resistance to *F. fujikuroi*, were reduced (Son et al., 2024a). Conversely, the *osnac101* mutant lines displayed reduced resistance to *F. fujikuroi*, indicating that OsNAC101 positively regulates resistance to the pathogen (Son et al., 2024a). Therefore, through targeted mutagenesis, Son et al. identified specific NAC transcription factors implicated in rice-pathogen interactions and elucidated how individual NAC genes influence the plant’s innate immune responses. Furthermore, this research showed that the CRISPR/Cas9 system is a powerful tool for generating elite rice cultivars that confer enhanced disease resistance through genome editing.

## Discussion

Traditional methods of mutagenesis, while useful, present challenges due to the time and labor required to generate and identify useful mutations. However, new plant breeding technologies (i.e., site-directed nucleases, oligonucleotide-directed mutagenesis, cisgenesis, intragenesis, RNA-dependent DNA methylation, grafting, reverse breeding, Agrobacterium-mediated infiltration, and synthetic genomics) have initiated a revolution in the field of plant breeding (Son and Park, 2022b). Especially, the amazing genome editing technology CRISPR is widely regarded as one of the most significant advancements in the history of biological science and technology. Recently studies using CRISPR/Cas9-mediated mutant libraries have led to significant advancements in crop breeding. DEG and CDPK mutant libraries have revealed key genes involved in insect pest resistance in cotton, particularly those related to calcium signaling pathways and protein kinases essential for immune responses (**Figure 1**). In rice, RLK and NAC mutant libraries have identified critical genes associated with bacterial and fungal pathogen resistance, providing insights into pathogen recognition and transcriptome reprogramming (**Figure 1**). These findings could potentially provide how crops are bred for biotic stress resilience.

While CRISPR/Cas9 technology has proven highly effective, there are still several challenges that need addressing (Mao et al., 2019; Son and Park, 2022a). One key challenge is the restrictive regulatory framework governing genetically modified organisms (GMOs), which hinders large-scale implementation, especially in regions where legal restrictions and public opposition to GMO crops persist. Therefore, further advancements in DNA-free genome editing techniques are essential to overcome these regulatory hurdles and facilitate broader adoption. Moreover, to date, there are many limitations on the application of this technique to various crops. Therefore, ongoing technological development is necessary to address these challenges. The advancement of genome-editing and transformation technologies and the screening of diverse mutant libraries will pave the way for a significant leap forward in crop development in the future.

CRISPR/Cas9 libraries have been established in a limited number of crop plants (Jacobs et al., 2017; Lu et al., 2017; Meng et al., 2017; Bai et al., 2020; Liu et al., 2020; Ramadan et al., 2021; Chen et al., 2022; Son et al., 2024a; Sun et al., 2024; Wang et al., 2024). Furthermore, the application of CRISPR/Cas9 library screening for the identification of immune-related genes has been realized only recently (Chen et al., 2022; Son et al., 2024a; Sun et al., 2024; Wang et al., 2024). These mutant libraries have been analyzed only in the context of specific pathogens. Therefore, further screening of these libraries across a range of biotic and abiotic stresses will reveal a wealth of genes involved in regulating stress responses. Broad-spectrum resistance is a highly valuable trait to incorporate into crop plants (Li et al., 2020; Koseoglou et al., 2022; Zhao et al., 2024). Continuous accumulation of results from CRISPR library screening for resistance to various pathogens will provide crucial insights into achieving broad-spectrum resistance in crops. Another challenge lies in understanding the complex molecular pathways governing plant immunity. While several key genes have been identified, the interplay between different genes and proteins is not always fully understood. Potential solutions include further research into plant-pathogen interactions and expanding the scope of CRISPR libraries to include genes with unknown functions, as demonstrated in cotton and rice studies.

The development of crops with enhanced resilience to biotic and abiotic stresses through CRISPR-based technologies presents significant potential for addressing global food security issues. As climate change increasingly affects agriculture, the capacity to engineer crops capable of withstanding extreme environmental conditions and pathogen pressures will be critical for sustaining agricultural productivity. Expanding CRISPR research to more plant species and traits, such as stress resilience or nutrient efficiency, could significantly contribute to the development of more sustainable agricultural practices. Moreover, advancements in CRISPR technology, such as the development of more efficient gene-editing pipelines will improve the speed and precision of gene identification and editing. This will enable more targeted breeding programs, resulting in crops that not only exhibit enhanced plant immunity but are also more nutritious and better adapted to evolving climate conditions.

Here, we presented an examination and analysis of a range of studies that have produced notable findings through CRISPR library screening, specifically aimed at the identification of novel genes implicated in enhancing crop immunity, thereby contributing to advancements within the broader scope of plant biology research. New gene-editing technologies are continuously being developed and advanced based on this remarkable technology. Therefore, the CRISPR library screening approach is an influential strategy for identifying new genes and advancing plant improvement. Despite challenges such as scaling the technology and navigating regulatory hurdles, the future prospects for CRISPR-mediated plant breeding are promising and have the potential to transform global agriculture.
